# Effectiveness of Posaconazole in Recalcitrant Fungal Keratitis Resistant to Conventional Antifungal Drugs

**DOI:** 10.1155/2014/701653

**Published:** 2014-08-11

**Authors:** A. Altun, S. A. Kurna, T. Sengor, G. Altun, O. O. Olcaysu, S. F. Aki, M. H. Simsek

**Affiliations:** ^1^Fatih Sultan Mehmet Education and Research Hospital, Clinic of Ophthalmology, 34752 Istanbul, Turkey; ^2^Department of Ophthalmology, Istanbul Bilim University, 34710 Istanbul, Turkey; ^3^Department of Pediatrics, Yeditepe University, 34752 Istanbul, Turkey; ^4^Erzurum Region Education and Research Hospital, Clinic of Ophthalmology, 25240 Erzurum, Turkey

## Abstract

*Purpose*. To present the success of posaconazole in two cases with recalcitrant fugal keratitis that were resistant to conventional antifungal drugs. *Method*. We presented two cases that were treated with posaconazole after the failure of fluconazole or voriconazole, amphotericin B, and natamycin therapy. Case 1 was a 62-year-old man with a history of ocular trauma. He had been using topical fluorometholone and tobramycin. His best corrected visual acuity (BCVA) was hand motion. He had 5.0 × 4.5 mm area of deep corneal ulcer with stromal infiltration. Case 2 was a 14-year-old contact lens user. He had been using topical moxifloxacin, tobramycin, and cyclopentolate. His BCVA was 20/200. He had a 4.0 × 3.0 mm area of pericentral corneal ulcer with deep corneal stromal infiltration and 2 mm hypopyon. *Results*. Both patients initially received systemic and topical fluconazole or voriconazole and amphotericin B and topical natamycin that were all ineffective. But the response of posaconazole was significant. After posaconazole, progressive improvement was seen in clinical appearance. BCVA improved to 20/100 in case 1 and 20/40 in case 2. *Conclusion*. Posaconazole might be an effective treatment option for recalcitrant fusarium keratitis and/or endophthalmitis resistant to conventional antifungal drugs.

## 1. Introduction

Fungal keratitis is a sight-threatening infection of the cornea that can lead to severe visual loss and even loss of the eye. It may be caused by several fungal pathogens and accounts for nearly 50% of all cases of infectious keratitis in developing countries [1, 2]. Fusarium species, especially Fusarium oxysporum and Fusarium solani, are the most fungal identified pathogens [3, 4]. They are found commonly in air and water [3]. Fusarium keratitis may be caused by ocular trauma with involvement of organic matter or may be related to contact lens wear or refractive surgery [5, 6].

Natamycin is a naturally occurring antifungal agent produced during fermentation by the bacterium* Streptomyces natalensis* and commonly found in soil. Natamycin is currently the preferred first-line drug for filamentous fungal keratitis. Because its corneal penetration is thought to be poor, its effectiveness in deeper keratitis is limited [7]. Amphotericin B is an amphoteric polyene macrolide that binds to ergosterol and alters the permeability of the cell by forming holes in the cell membrane. Triazoles (voriconazole, fluconazole, itraconazole, and posaconazole) inhibit the biosynthesis of ergosterol, an essential component of the fungal cell wall.

Voriconazole, fluconazole, amphotericin B, and natamycin are the conventional antifungal agents, which are well known options announced to be successful in fungal keratitis in many studies [8–10]. But fungal keratitis resistant to these drugs had been also reported in literature [11, 12]. Posaconazole is a synthetic structural analog of itraconazole and has activity* in vitro* against yeasts and filamentous fungi, including the agents of mucormycosis [13]. Posaconazole has been used successfully to treat fungal keratitis either as a standalone therapy or in conjunction with other antifungal agents [11, 14]. In this study, we would like to present two patients treated successfully with posaconazole for recalcitrant fungal keratitis, who were resistant clinically to voriconazole, fluconazole, amphotericin B, and natamycin.

## 2. Methods

We identified two patients from two different centers who had resistant fungal keratitis. The diagnosis of fungal infection was made according to clinical evaluation and positive cytologic examination of the fungus. Each case was reviewed for mechanism of inoculation, including trauma or contact lens wear or previous use of medications. Each patient underwent a detailed clinical evaluation that included recording of medical history, Snellen visual acuity testing, and slit-lamp biomicroscopy. Corneal scrapings were obtained under topical anesthesia (0.5% proparacaine hydrochloride: Alcaine; Alcon, Puurs, Belgium) and were sent for microbiological investigation including potassium hydroxide wet-mount preparation, Gram smear, and cultures on blood agar, chocolate agar, and Sabaroud agar. 

**Figure 1:**
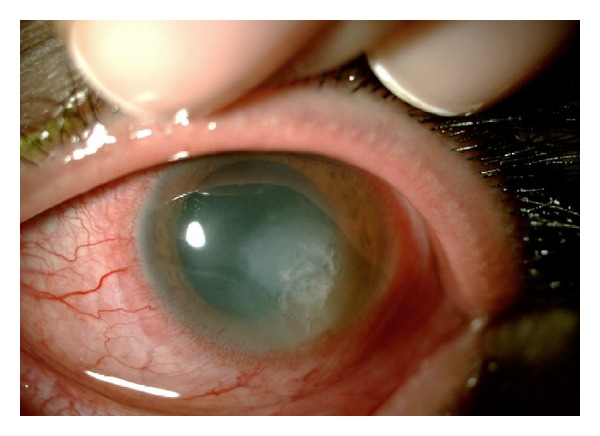
The right eye of the case 1 with deep corneal ulcer and stromal infiltration.

*Case 1.* A 62-year-old man presented from rural Turkey with a 7-day history of redness and stinging involving the right eye without associated pain. He provided a history of nonpenetrating ocular trauma with organic material (bough) 10 days prior to presentation. He had been using topical 0.1% fluorometholone (FML, Allergan, France) and 0.3% tobramycin (Tobrex; Alcon, Puurs, Belgium) eye drops, both every 3 hours for the preceding week. There was no history of contact lens use, previous ocular surgery, or concurrent systemic disease. His reported occupation was farmer and kept a garden nearby his home. Before presentation, various combinations of 0.3% lomefloxacin (Okacin; Novartis, Hettlingen, Switzerland), 0.3% gatifloxacin (Zymar; Allergan, Texas, USA), polymyxin B-oxytetracycline (Terramycin; Pfizer, Istanbul, Turkey), and tropicamide 0.5% (Tropamid; Bilim, Istanbul, Turkey) were tried without improvement for a week. His best corrected visual acuity was hand motion in the right eye (pinhole visual acuity) and 20/20 in the left eye. Intraocular pressure was approximately 20 and 14 mm Hg in the right eye (digital palpation the cornea) and left eye (Goldmann applanation tonometry), respectively. On slit-lamp biomicroscopic examination of the right eye, a 5.0 × 4.5 mm area of deep corneal ulcer and stromal infiltration with a clear corneal periphery was evident and no hypopyon. There was 5–10 per cells per high-powered field in the anterior chamber (Figure 1). Dilated fundus examination was unremarkable. The left eye appeared normal.

**Figure 2:**
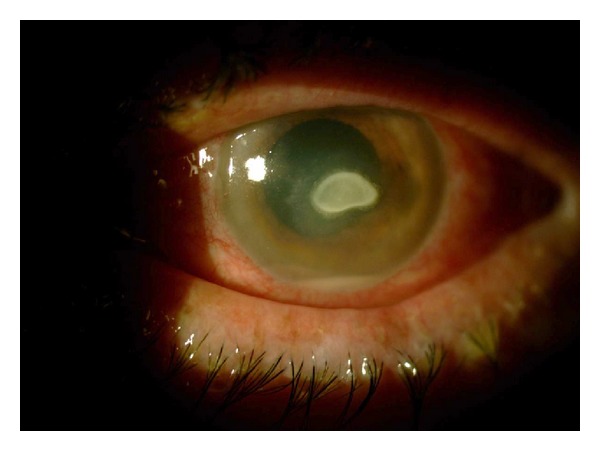
The right eye of the case 2 with paracentral corneal ulcer accompanying corneal stromal infiltration and hypopyon.

*Case 2.* A 14-year-old boy was referred to our clinic with a 4-day history of redness, stinging, and a burning sensation involving the right eye. He was a soft contact lens wearer in a daily schedule. There was no history of ocular trauma, previous ocular surgery, or concurrent systemic disease, but diagnosis of diabetes mellitus type I had been made recently. Before presentation, various combinations of 0.5% moxifloxacin (Vigamox; Alcon, Texas, USA), 0.3% tobramycin (Tobrex; Alcon, Puurs, Belgium), and subsequently cyclopentolate 1.0% (Sikloplejin; Abdi-Ibrahim, Istanbul, Turkey) eye drops were tried without improvement. His BCVA was 20/200 in the right eye (pinhole visual acuity) and 20/20 in the left eye. On slit-lamp biomicroscopic examination, a 4.0 × 3.0 mm area of paracentral corneal ulcer accompanying deep corneal stromal infiltration was evident with associated 2 mm hypopyon (Figure 2). Ultrasound examination revealed organization of opacities in the vitreous that were giving rise to thought endophthalmitis. The hypopyon might be sterile because of relatively good visual acuity and normal appearing fundus examination. The left eye was normal, and the fundus examination showed no pathology bilaterally.

At the time of presentation, both cases was started on multiple antifungal agents including topical natamycin 0.5% ointment (Pima-Biciron, S & K Pharma) 4 times daily, and systemic and topical (0.5%, hourly) amphotericin B. Topical and systemic triazols (fluconazole for the case 1, voriconazole for the case 2) were also added to regimen. Voriconazol (Vfend; Pfizer, Ireland) was used 200 mg twice a day orally, and 1.0% prepared eyedrops hourly. Fluconazole (Diflucan, Pfizer, New York, USA) was used 200 mg once a day orally, and 0.3% prepared eye drops hourly. Topical drops were prepared aseptically with 0.9% sodium chloride, with 48 hours' expiry. Corneal and epithelial debridement were performed twice to enhance the penetration of the antifungal drugs and to accelerate the healing process. Cyclopentolate was administered topically 5 times a day to relieve occurring pain and to prevent possible intraocular synechia.

## 3. Results

The cause of infection was ocular trauma with organic matter in case 1, and contact lens wear in case 2. Both patients had been treated for presumed bacterial infection before presentation, case 1 with the addition of topical corticosteroid. Topical natamycin and conventional systemic antifungal drugs (amphotericin B, voriconazole, and fluconazole) were ineffective as well. None of the patients required penetrating keratoplasty. Case 1 had just fungal keratitis, but case 2 had also possible associated endophthalmitis. During the course of treatment, the patients developed elevated liver function tests (LFTs) that thought to be possibly related to voriconazole and posaconazole. Discontinuation of the drug was not needed during the course and the LFTs resolved after the drug was discontinued.

Corneal scrapings were processed for microbiological investigation and cytological examination. Cultures and smears returned negative, but the cytological examination revealed fungal hyphae (Fusarium) for both cases.

**Figure 3:**
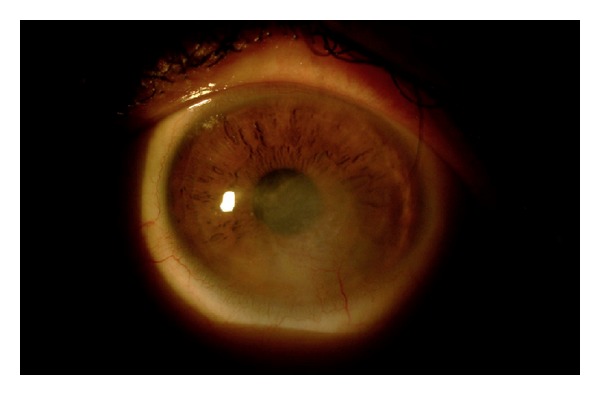
Resolved ulceration and regressed corneal vascularization after posaconazole.

Despite conventional antifungal therapy (natamycin, voriconazole, fluconazole, and amphotericin B), the ulceration continued, and the cornea around the lesion began thinning, and the patients had worsening pain. Oral (200 mg suspension 4 times daily) and topical posaconazole (Noxafil, Schering Plough) was started on day 12 of treatment because of the lack of clinical improvement. Topical application of posaconazole was made with the same suspension (4 mg/0.1 mL) hourly. The response to topical and systemic posaconazole was remarkable. Progressive improvement was seen in clinical appearance in 4 days for case 1 and 5 days for case 2. The ulcerations had completely resolved, the conjunctival hyperemia had disappeared, and the corneal vascularization had regressed in 2 weeks; however, central corneal hazes were still present (Figure 3).

## 4. Discussion

Fungal keratitis is usually misdiagnosed as bacterial keratitis because isolation of the pathogen is difficult and fungal growth in culture requires time. Delayed diagnosis and possible history of steroid application may contribute worse clinical outcome [15].

Treatment of fungal keratitis remains problematic, in part because of the expanding list of fungal pathogens and in part because of the relatively short list of available therapeutic agents. Poor bioavailability and limited ocular penetration, especially in deeper lesions, are the main limitations of the antifungal agents [7]. Corneal and epithelial debridement and intrastromal injection are the surgical techniques that might let the drug penetrate into the cornea to treat deep infections [8]. We performed corneal and epithelial debridement, without intrastromal injection to avoid the risk of corneal perforation.

Fungal keratitis may be caused by ocular trauma with involvement of organic matter, or may be related to contact lens wear or refractive surgery. One of our cases (case 1) had a history of ocular trauma, possibly with organic matter. The other case (case 2) was an inappropriate wearer of contact lens. Contact lenses and their solutions are often known as a risk factor for Acanthamoeba keratitis, but they had been also reported to a risk factor for fungal keratitis [5, 6].

The commonly available antifungal agents are natamycin, amphotericin B, fluconazole, itraconazole, and voriconazole. Natamycin is a polyene, and the only approved and commercially available topical antifungal. It has good efficacy against filamentous fungi but with low ability to penetrate into the cornea. On the basis of literature, natamycin appears to be the initial drug of choice for Fusarium keratitis, but its effectiveness in deeper keratitis is limited.* In vitro* studies suggest that Fusarium is most consistently sensitive to amphotericin B, but several clinical failures have been also reported. We initially used natamycin and amphotericin B at the time of diagnosis and resumed for 12 days with no clinical improvement.

Triazoles inhibit the biosynthesis of ergosterol, an essential component of the fungal cell wall. Voriconazole is a triazole antifungal agent derived from fluconazole with activity against various fungi [16]. Voriconazole has been shown to have a broad spectrum of activity against nonocular isolates of Aspergillus species, Candida species, and Fusarium species [16, 17]. Voriconazole is a potent triazole with 100%* in vitro* susceptibility reported in common for the ocular fungal pathogens, compared to only 60–82.4% for fluconazole, itraconazole, amphotericin B, and ketoconazole [3]. Voriconazole has excellent* in vitro* activity against Candida and Aspergillus species is known to be resistant to amphotericin B, fluconazole, and itraconazole [18]. Voriconazole has been used successfully to treat fungal keratitis either as a standalone topical therapy [9, 10] or with systemic administration [7]. Although voriconazole is the drug of choice in recalcitrant resistant fungal keratitis, there have been also reported cases that were resistant to voriconazole. Elmer et al. reported 2 culture proven Fusarium keratitis which was resistant to voriconazole but sensitive to posaconazol [11].

Posaconazole (Noxafil, Schering-Plough) oral suspension was approved in the fall of 2006.* In vitro* and* in vivo* studies have shown that it has broad-spectrum activity against nonocular isolates of most Candida species, Cryptococcus neoformans, Aspergillus species, zygomycetes, and endemic fungi [19]. Posaconazole has also been reported to be an effective agent against Fusarium keratitis [11, 14]. Cuenca-Estrella et al. showed posaconazole to be active against a majority of isolates resistant to fluconazole and itraconazole [20]. Tu et al. reported three cases of Fusarium ocular infections [11] resistant to amphotericin B and voriconazole that were treated with posaconazole. Although* in vitro* studies indicate variable activity against Fusarium species, posaconazole has been found to retain activity against isolates resistant to voriconazole and to have greater clinical efficacy than voriconazole [19, 21]. Initially, we also tried to treat our two cases with systemic and topical voriconazole or fluconazole and amphotericin B. Because of the unresponsiveness to these drugs, we switched to posaconazole. The response of our patients' infection to topical and systemic posaconazole was remarkable. This might be because of its relatively more lipophilicity that enhances its ability to penetrate ocular tissues easier [14].

Herein, we would like to report the results of using oral and topical posaconazole showing good clinical efficacy in 2 eyes with recalcitrant fungal keratitis non-responsive to voriconazol, fluconazole, amphotericin B, and natamycin. Posaconazole should be considered in mind in the treatment of the Fusarium keratitis and/or endophthalmitis in cases resistant to standard antifungal therapy.
